# Supplementary material for: Electronic structure blurring-mediated solid-state H2O2 electrosynthesis with high productivity
Source: Nat Commun. 2025 Nov 24;16:10360. doi: 10.1038/s41467-025-65335-5 (PMC12645009; doi:10.1038/s41467-025-65335-5)
Supplement: Supplementary file 1 — Supplementary Information [file 41467_2025_65335_MOESM1_ESM.pdf]

1. Supplementary Methods

1.1 Computational methods

DFT calculations were performed by the MedeA-Vienna *ab initio* Simulation Package (VASP). The Perdew-Burke-Ernzerhof (PBE) generalized gradient approach was used to define the exchange-correlation potential[1, 2]. The interaction between the atomic cores and electrons was described by using projector augmented wave method (PAW)[3, 4]. The plane wave energy cutoff was set to be 400 eV. The Brillouin zone in the real space was sampled with a 1 × 1 × 1 Monkhorst-Pack K-point grid. The convergence criterion was set to be 10^{-5} eV and 0.02 eV/Å for energy and force in the geometry optimizations, respectively. A Gaussian smearing method was employed with 0.05 eV width. Hubbard-U correction method (DFT+U) was carried out to improve the description of highly correlated Zn 3d orbitals with the value of U-J set to be 2.5 eV. The vacuum layer is set to be 15 Å to avoid interaction between two neighboring images.

The detailed Gibbs free energy has been calculated according to the following equation:

$$G = E + ZTE - TS \tag{1}$$

Where *G*, *E* and *ZTE* refer to chemical Gibbs free energy, electronic energy and zero-point energy, respectively. The entropy can be calculated by the sum of the vibrational, rotational, translational, and electronic contribution as to:

$$S=S_v+S_r+S_t+S_e \tag{2}$$

Since $S_e \approx 0$ at the fundamental electronic level.

For the case of solids and adsorbates, some approximations can be adopted: Translational and rotational motions can be omitted, therefore, $S_t \approx 0$ and $S_r \approx 0$. In this case, all the entropy values come from the vibrational contribution: $S = S_v$

Finally, Gibbs free energy for different states was calculated as to:

$$G = E + ZTE - TS_v \tag{3}$$

1.2 Economic calculation

In order to clearly demonstrate the cost of the solidified H_2O_2 electrolysis system, a techno-economic evaluation was performed. we assumed a cell operation of 200 mA cm^{-2}, which has an cell voltage of 3.85 V. Assuming a capture capacity of 1 ton-KF·H_2O_2 day^{-1}, the total current needed is:

$$\begin{aligned} Total\ current\ &= 1000\ \frac{kg}{day} * \frac{day}{24\ h} * \frac{h}{3600s} * \frac{kmol}{92\ kg} * 2e^{-} * 96485\ \frac{C}{mol} * \frac{1}{89.62\%} \\ &= 27088.4\ A \end{aligned} \tag{4}$$

The electrolyzer area needed is the total current divided by the current density:

$$Total\ electrolyzer\ area\ = \frac{27088.4\ A}{0.21875\ \frac{A}{cm^2}} * \frac{m^2}{10^4 cm^2} = 12.38\ m^2 \tag{5}$$

The power needed is given from P=UI, the cell voltage is 4.52 V for 200 mA cm^{-2}:

$$Power\ = Voltage * 27088.4\ A * \frac{W}{10^3\ kW} = \ 3.85 * 27088.4\ = 104.3\ kW \tag{6}$$

Electrolyzer cost

From the DOE H_2A analysis for central grid electrolysis, the electrolysis cost for the stack component is \$1619 m^{-2}. So we set the instillation factor as 1.2. Consequently, the cost for the reference electrolysis is:

$$Electrolyzer\ \cos t\ = \ 12.38\ m^2 * \frac{\$1619}{m^2} * 1.2 = \ \$24051.9 \tag{7}$$

Herein, the capital recovery factor (CRF) is based on a discount rate (denoted as i; we use 5% for all the CRF calculations) and the material lifetime (40 years).

$$CRF_{electrolyzer} = \frac{i(1+i)^{year}}{(1+i)^{year}-1} = \frac{0.05*1.05^{40}}{1.05^{40}-1} = 0.058 \quad (8)$$

$$Electrolyzer\ \ cost_{per\ ton\ KF\cdot H_2O_2} = \$24051.9*\frac{0.058}{350} = \$4/day \quad (9)$$

Balance of plant cost

From the H2A, the balance of plant capital cost is 35% of the total cost, while the stack is 65%:

$$Bop\ capital\ \ cost = \$24051.9*\frac{0.35}{0.65} = \$12951 \quad (10)$$

Herein, the capital recovery factor (CRF) is based on a discount rate (denoted as i; we use 5% for all the CRF calculations) and the material lifetime (40 years).

$$Bop\ capital\ \ cost_{per\ ton\ KF\cdot H_2O_2} = \$12951*\frac{0.058}{350} = \frac{\$2.1}{day} \quad (11)$$

Therefore, the overall capital cost is \$6.1 per ton $KF\cdot H_2O_2$.

Electricity cost

The electricity cost is calculated from the power requirement and the price of electricity, assuming the electricity price is 5 cent kWh^{-1}:

$$Electricity\ \ cost_{per\ ton\ KF\cdot H_2O_2} = 104.3\ kW*24\ hr/day*\frac{\$0.05}{kWh} = \frac{\$125.2}{day} \quad (12)$$

Input chemicals cost

For the input chemicals cost, we account for the cost from the consumed H_2O for OER reaction. The water price is estimated as \$ 0.003 per liter.

$$H_2O\ flow\ rate\ =\ 27088.4\ A*\frac{1}{4e^{-}*96485\ \frac{C}{mol}}*\frac{0.018\ kg}{mol}*\frac{86400\ s}{day} = 109.2\ \frac{kg}{day} \quad (13)$$

$$\text{Cost of consumed } H_2O\ =\ \$\ 0.003*109.2 = \$\ 0.3/day \quad (14)$$

Consider 2.5% loss for KF ($1800/ton) chemicals,

$$KF\ mass\ flow\ rate\ =\ 27088.4\ A*\frac{1}{1*96485\ \frac{C}{mol}}*\frac{0.058\ kg}{mol}*\frac{86400\ s}{day} = 1406.9\ \frac{kg}{day} \quad (15)$$

$$\text{Cost of consumed } KF\ =\ 1406.9\ \frac{kg}{day}*0.025*\frac{\$\ 1.8}{kg} = \$\ 63.3/day \quad (16)$$

Stack replacement cost

For the stack replacement cost, assuming 30% of the uninstall cost for 7 years[5, 6].

$$\text{Stack replacement cost}\ =\ \$\ 24051.9*\frac{0.30}{7*350} = \$\ 2.9/day \quad (17)$$

Other operational costs

Other operational costs, such as labor and maintenance, are assumed to be 2.5% of the electrolyzer cost per ton of KF·H_2O_2[7].

$$\text{Other cost}_{per\ ton\ KF\cdot H_2O_2} = \$\ 24051.9*\frac{0.025}{350} = \$\ 1.7/day \quad (18)$$

Thus, the overall cost to regenerate 1 ton KF·H_2O_2/day using bicarbonate is:

$$\text{Overall cost}_{per\ ton\ KF\cdot H_2O_2} = \$6.1+\$125.2+\$63.3+\$0.3+\$2.9+\$1.7 = \$199.5 \quad (19)$$

Accordingly to the H_2O_2 gravimetric density of 36.9 wt% in KF·H_2O_2, the capital cost of H_2O_2 is 0.539 kg^{-1}, which is much smaller than industrial anthraquinone oxidation method ($1.5 kg^{-1}).

1.3 Calculations of theoretical limit of 2e-ORR

To further investigate the effect of O_2 partial pressure on activities, the 2e-ORR performance of ZIF-350 were tested at lower O_2 partial pressures (21% ~ 5%, Supplementary Figs. 24−26). The limiting diffusion current densities are calculated for different concentration mixtures across the electrode surface according to the following equation[8]:

$$\mathrm{I_d} = \frac{nFD_{ON}\Pi}{\delta} In \frac{\Pi}{c_N}$$

The limiting diffusion current density (I_d) (mA cm^{-2}) is determined by the gas reactant flow (∏) (mol cm^{-3}), the diffusion coefficient of oxygen in natural air (D_{ON}) (cm^2 s^{-1}), and the concentration of nitrogen on the surface of the gaseous diffusion layer (c_N) (mol cm^{-3}). Additionally, the electron reaction numbers (n), the Faraday constant (F) (96485 C mol^{-1}), and the thickness of the gaseous diffusion layer (δ) (cm) are also considered.

Obviously, the limiting diffusion current densities of the reaction decrease continuously with O_2 partial pressures. Notably, The ZIF-350 catalyst can maintain the current density close to the theoretical limits under different partial pressures (Fig. 2f).

3. Supplementary Figures

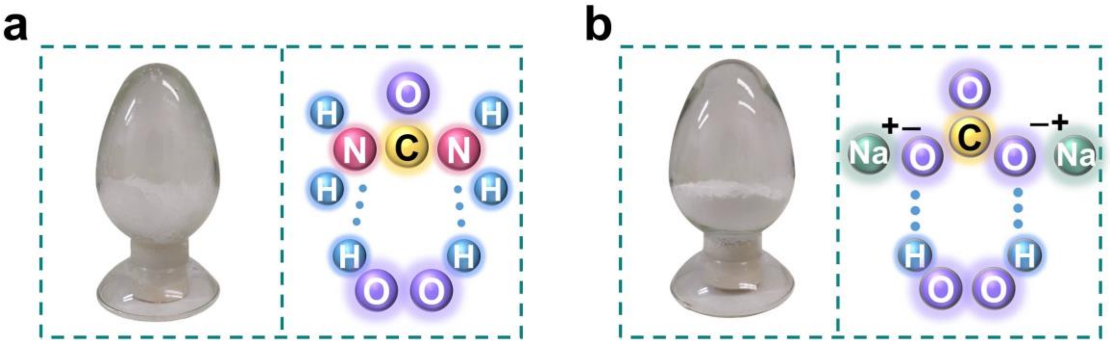

Supplementary Figure 1. Optical image and structural illustration of urea peroxide and sodium percarbonate. a, $CO(NH_2)_2 \cdot H_2O_2$. **b**, $Na_2CO_3 \cdot 1.5H_2O_2$.

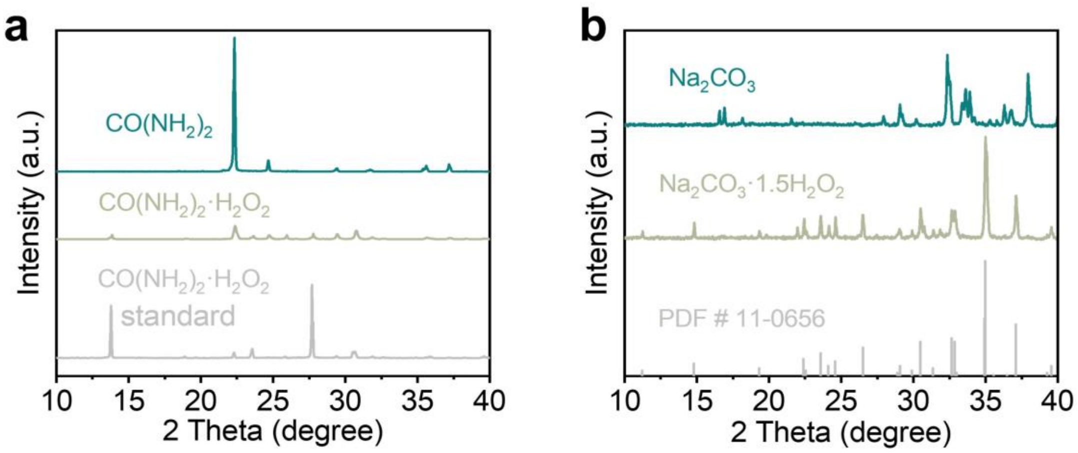

Supplementary Figure 2. XRD patterns of urea hydrogen peroxide and sodium percarbonate. **a**, $CO(NH_2)_2$ and $CO(NH_2)_2{\cdot}H_2O_2$. **b**, Na_2CO_3 and $Na_2CO_3{\cdot}1.5H_2O_2$.

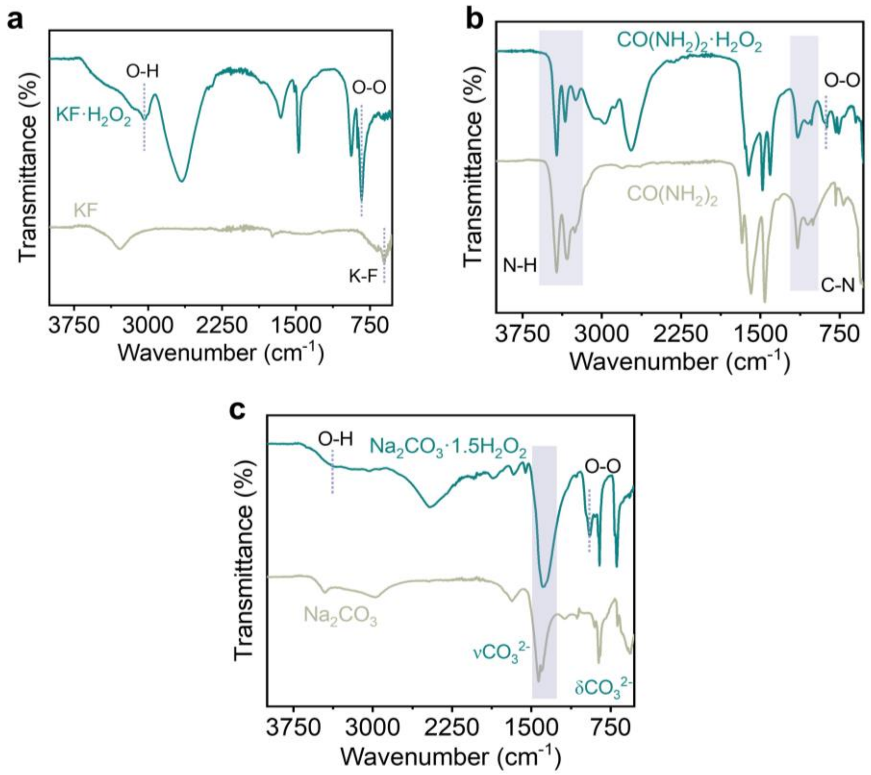

Supplementary Figure 3. FTIR spectra of peroxosolvates. **a**, KF and $KF{\cdot}H_2O_2$. **b**, $CO(NH_2)_2$ and $CO(NH_2)_2{\cdot}H_2O_2$. **c**, Na_2CO_3 and $Na_2CO_3{\cdot}1.5H_2O_2$. Generally, KF alone only show typically K-F vibration at 603 cm^{-1} and O-H at 3292 cm^{-1}. While for $KF{\cdot}H_2O_2$, both vibrations have changed significantly, *i.e.*, 571 cm^{-1} for K-F and 3036 cm^{-1} for O-H vibration. The peak shift might be originated to the formation of O-H··F bonds. The same phenomenon has been observed for $CO(NH_2)_2{\cdot}H_2O_2$ and $Na_2CO_3{\cdot}1.5H_2O_2$ counterparts.

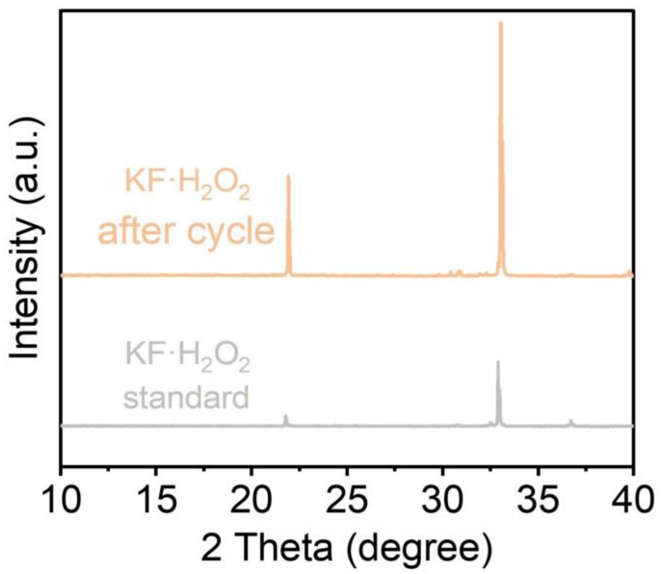

Supplementary Figure 4. XRD patterns of potassium fluoride hydrogen peroxide ($KF{\cdot}H_2O_2$) after 100 storage-release cycles.

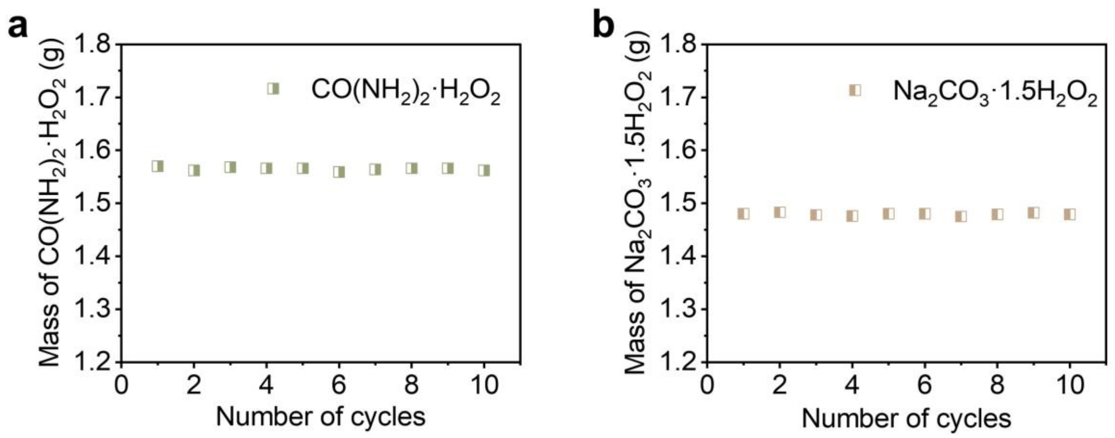

Supplementary Figure 5. Mass of $CO(NH_2)_2{\cdot}H_2O_2$ and $Na_2CO_3{\cdot}1.5H_2O_2$ after 10 storage-release cycles.

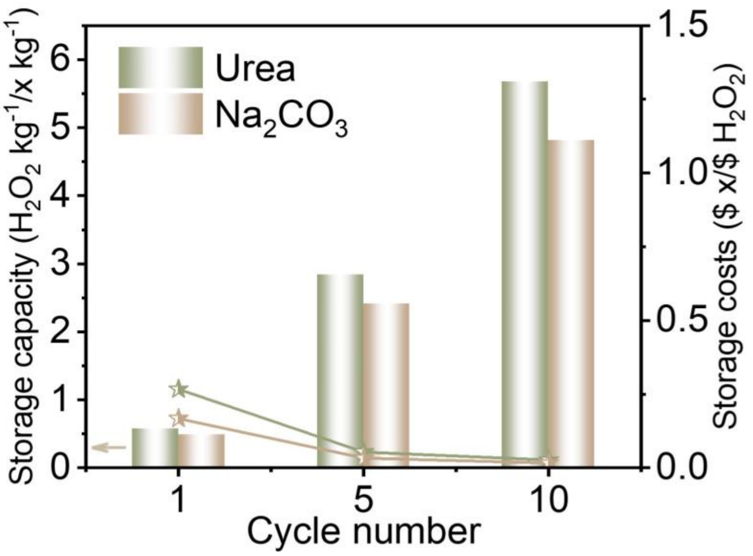

Supplementary Figure 6. Changes in H_2O_2 storage capacity and cost by urea and Na_2CO_3 for 10 storage-release cycles. Where x stands for urea and Na_2CO_3.

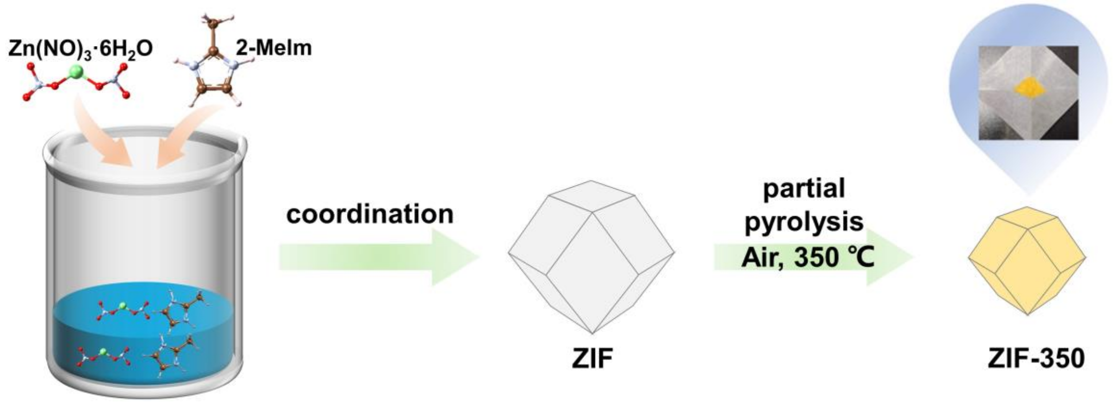

Supplementary Figure 7. Schematic diagram of the synthesis of ZIF-350.

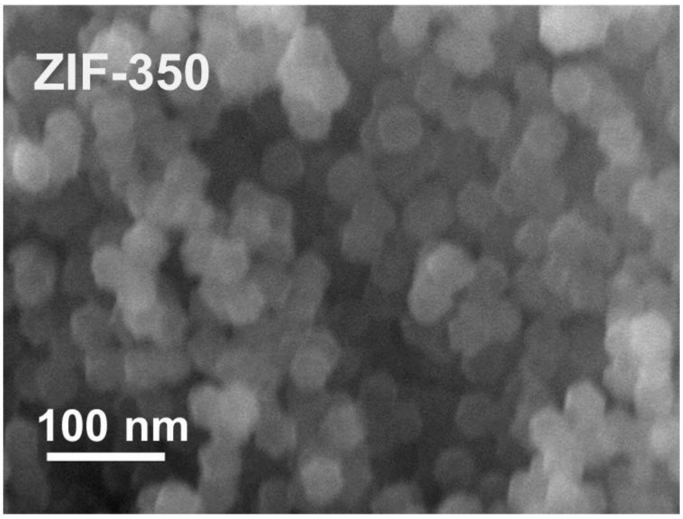

Supplementary Figure 8. The SEM image of ZIF-350.

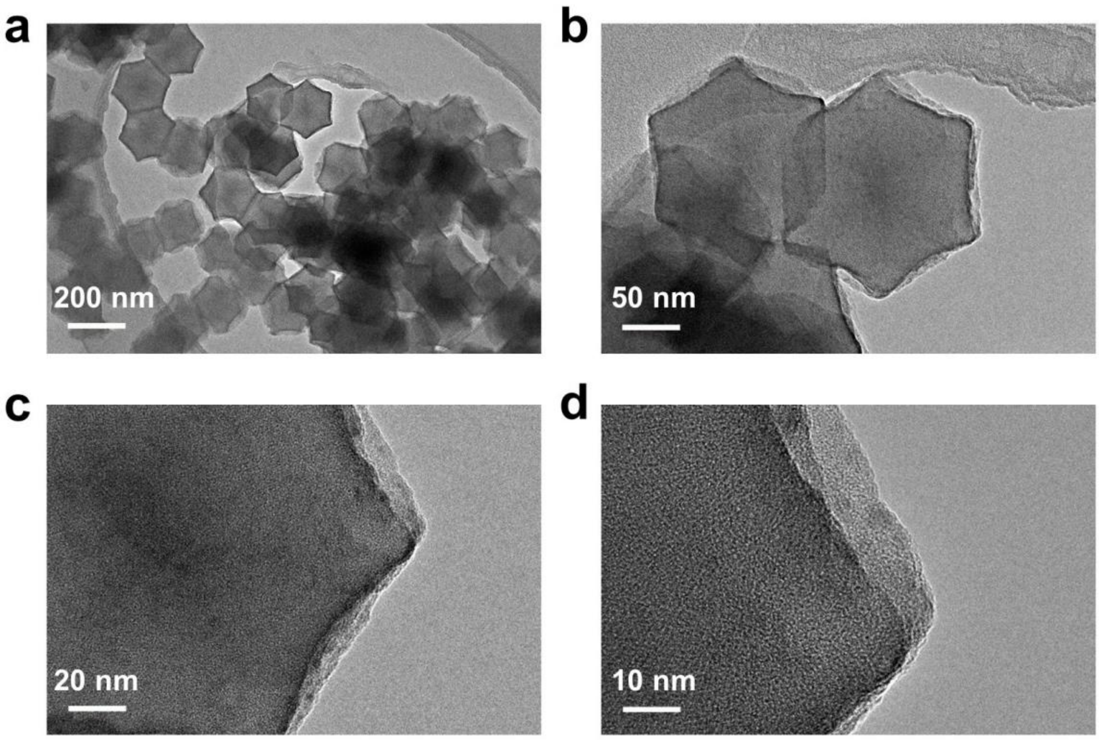

Supplementary Figure 9. Additional TEM images of ZIF-350. The TEM images illustrate that ZIF-350 possesses a rhombic dodecahedral structure with particle sizes ranging from 100 to 200 nm.

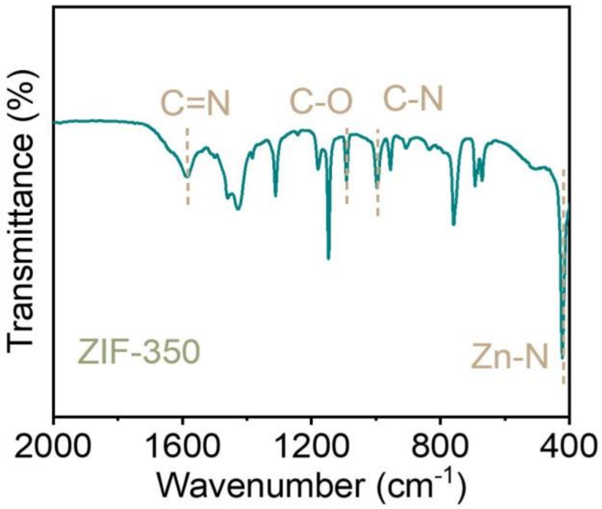

Supplementary Figure 10. The Fourier infrared spectra (FT-IR) of ZIF-350. The FT-IR spectra shows ZIF-350 retaining the main backbone structure of pristine ZIF, with a few characteristic peak intensities significantly weakened. This is due to the partial breakage of the Zn-N bond caused by low-temperature pyrolysis.

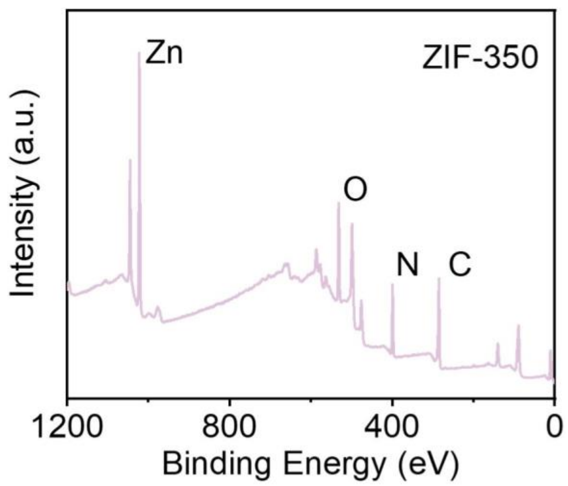

Supplementary Figure 11. The XPS survey of ZIF-350.

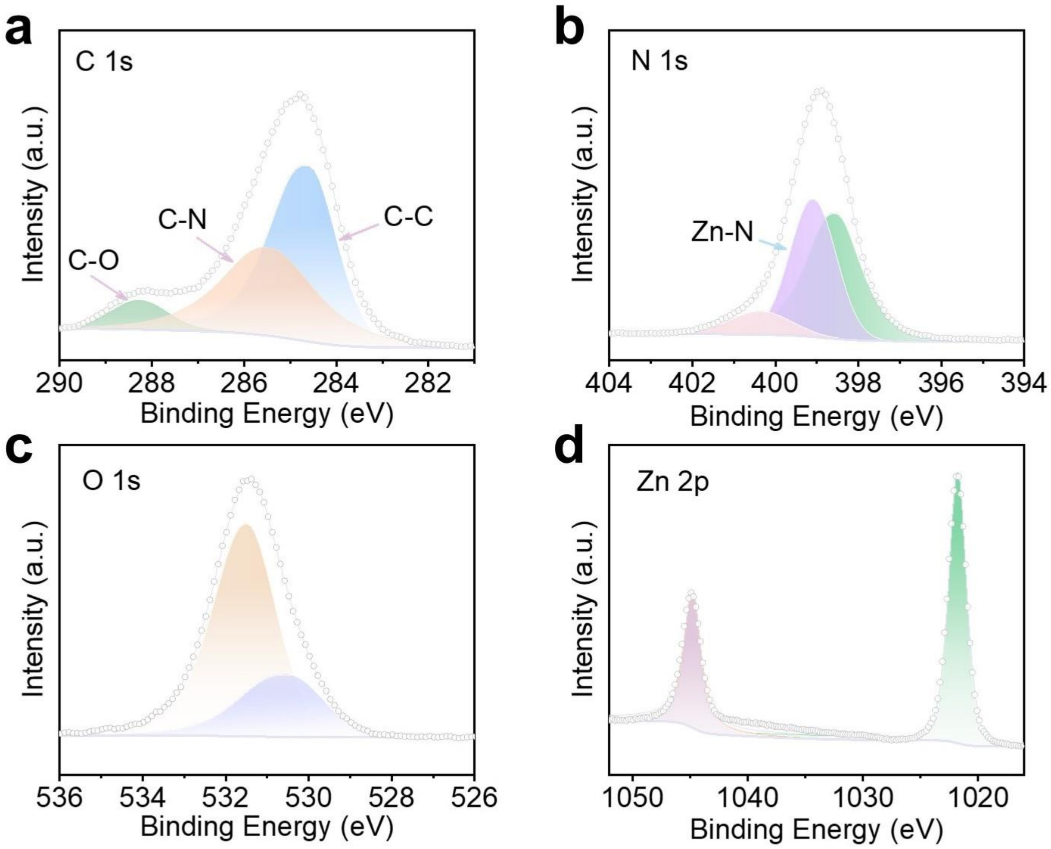

Supplementary Figure 12. The XPS analyses of ZIF-350. a, C 1s. **b,** N 1s. **c,** O 1s and **d,** Zn 2p. The C 1s XPS spectrum of ZIF-350 can be effectively resolved into distinct peaks of C=C (284.6 eV), C-N (285.5 eV) and C-O (288.2 eV), suggesting partial oxidation of the carbon skeleton introducing a small amount of oxygen-containing groups.

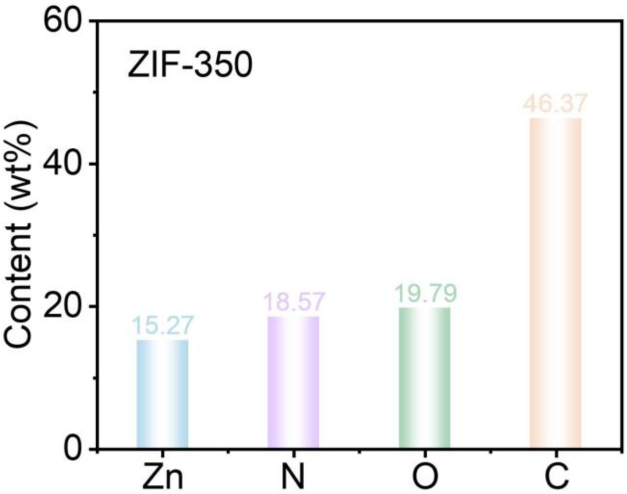

Supplementary Figure 13. The percentages of Zn, N, O, and C elements in ZIF-350 measured by XPS. In accordace to the partial collapse of the ZIF skeleton in ZIF-350 after low-temperature pyrolysis, the percentages of Zn and O elements in ZIF-350 increase while the percentages of C and N elements decrease. This allows for more Zn active sites to be exposed to promote 2e-ORR. Further, several recent reports have also confirmed that the introduction of oxygen-containing groups can effectively enhance 2e-ORR activity[9, 10].

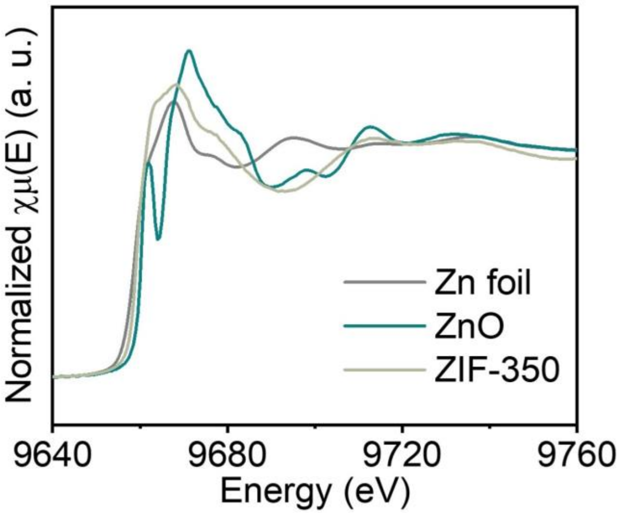

Supplementary Figure 14. X-ray absorption near edge structure (XANES) spectra of ZIF-350, Zn foil and ZnO. The K-edge X-ray absorption near edge structure (XANES) spectrum of Zn reveals that the absorption edge of ZIF-350 lies between those of Zn foil and ZnO, suggesting the valence state of the Zn material in the sample ranging between 0 and +2.

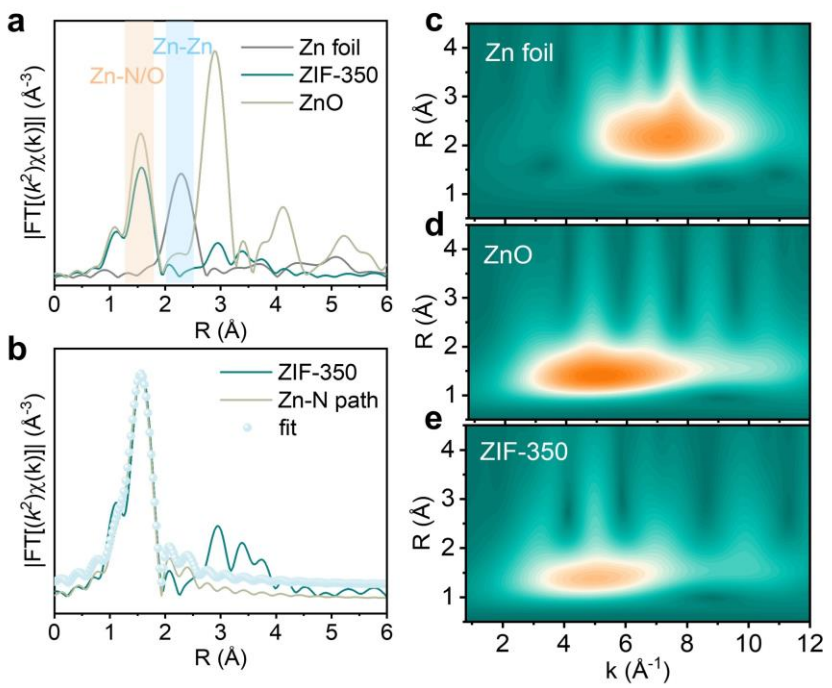

Supplementary Figure 15. Extended X-ray absorption fine structure (EXAFS) spectra and wavelet transform(WT)-EXAFS contour plots of ZIF-350, Zn foil and ZnO. a, EXAFS curve of ZIF-350, Zn foil and ZnO. **b,** Fitting curves of EXAFS spectra of ZIF-350. **c–e,** The corresponding wavelet transform (WT)-EXAFS contour plots of ZIF-350, Zn foil and ZnO.

Supplementary Note 1:

In the Fourier-transformed (FT) R-spaced EXAFS spectrum, ZIF-350 displays a prominent peak at approximately 1.5 Å corresponding to the Zn-N path. This finding provides additional evidence that the sample maintains an organic framework structure. The wavelet transform (WT) contour map reveals an intensity maximum at approximately 5 Å indicative of Zn-N coordination (Supplementary Fig. 15 e). This is quite different from the results for Zn foil and ZnO. Synchrotron radiation results suggest that the Zn species in ZIF-350 are predominantly present in the form of $Zn\text{-}N_2$.

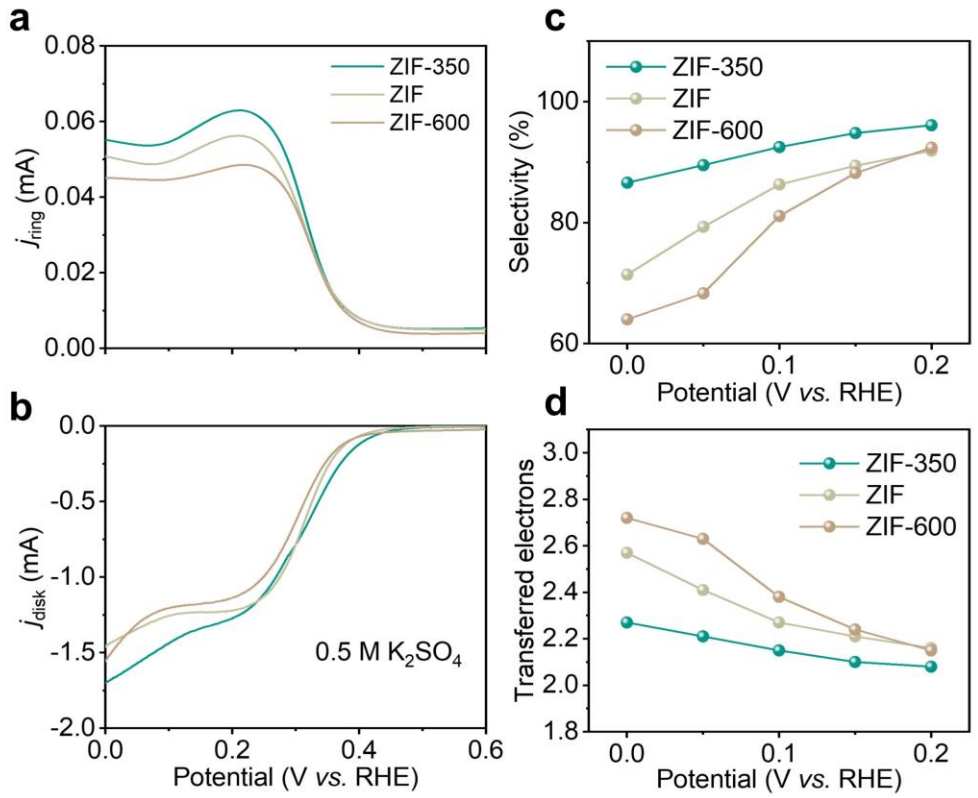

Supplementary Figure 16. Rotating ring-disk electrode (RRDE) test of ZIF-350, ZIF and ZIF-600 in 0.5 M K_2SO_4 under O_2. a, b RRDE linear sweep voltammetry (LSV) of ZIF-350, ZIF and ZIF-600 recorded at the rotation rate of 1600 rpm (lower panel), along with the detected H_2O_2 currents on Pt ring electrode (upper panel). **c,** H_2O_2 selectivity calculated on the basis of RRDE. **d.** Electron transfer number (n) calculated on the basis of RRDE.

Supplementary Note 2:

The intrinsic ORR activity of the three catalysts was investigated in a typical rotating ring-disk electrode (RRDE) system using a three-electrode system in O_2 saturated 0.5 M K_2SO_4 (pH ~ 7). Impressively, ZIF-350 catalyst displays better 2e-ORR intrinsic activity with a more positive onset potential (defined as the potential at the ring current density of -0.1 mA cm^{-2}) and higher H_2O_2 oxidation current density

than other samples (Supplementary Figs. 16 a, b). The number of transferred electrons and the H_2O_2 selectivity as a function of potential are shown in Supplementary Figures 16 c, d, as an important indicator for the assessment of 2e-ORR activity. In a wide window of potential ranging from 0 to 0.2 V (*vs.* RHE), ZIF-350 electrode discloses an excellent H_2O_2 selectivity (96%), superior to ZIF and ZIF-600. The electron transfer numbers (N) of ZIF-350 catalyst (2.08 ~ 2.27) is closer to 2 than that of ZIF (2.16 ~ 2.57) as well as ZIF-600 (2.15 ~ 2.72), thus indicating the outstanding 2e-ORR activity of ZIF-350.

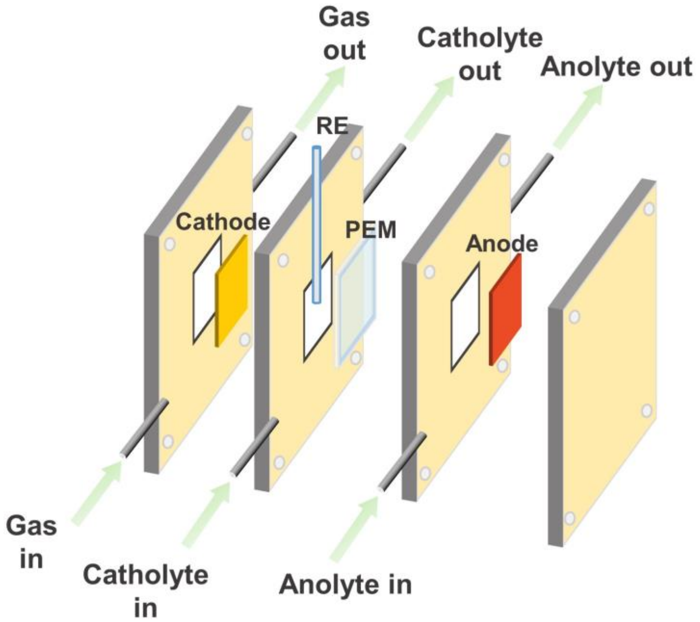

Supplementary Figure 17. Schematic diagram of the flow-type cell for H_2O_2 production.

Supplementary Note 3:

To achieve ultrafast 2e-ORR under industrial-level current densities, a flow electrolytic cell system was assembled. As shown in Supplementary Figure 17, the microporous layer loaded with catalysts is placed on one side of the gas diffusion electrode (GDE), while the electrolyte circulates through cathode compartment. On the other side is the gas flows.

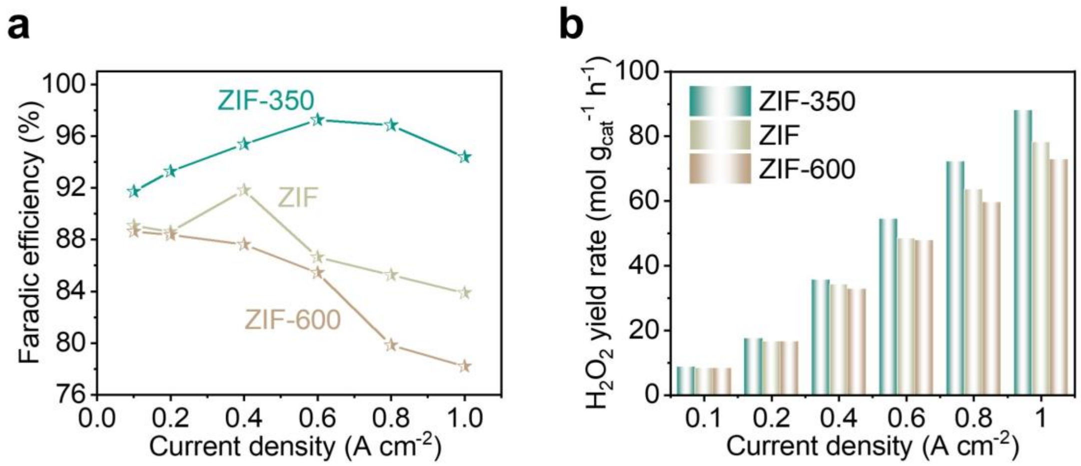

Supplementary Figure 18. The ORR performances of ZIF-350, ZIF and ZIF-600 in 0.5 M K_2SO_4 under O_2 in flow cells. a. H_2O_2 Faradaic efficiencies. **b.** H_2O_2 yield rates.

Supplementary Note 4:

Three electrodes were subjected to chronoamperometry tests to evaluate the 2e-ORR performance of the catalysts at different current densities. With the current density increasing from 0.1 to 1.0 A cm^{-2}, the Faradaic efficiencies of ZIF-350 electrode are maintained over 90% (91.72% ~ 94.38%) and the yield of H_2O_2 almost linearly increases from 8.66 to 87.97 mol g_{cat}^{-1} h^{-1} (Supplementary Fig. 18 a). In sharp contrast, ZIF and ZIF-600 only provide poor Faradaic efficiencies (83.88% and 78.2%) and inferior yield rates of H_2O_2 (49.93 and 48.69 mol g_{cat}^{-1} h^{-1}) at 1.0 A cm^{-2}.

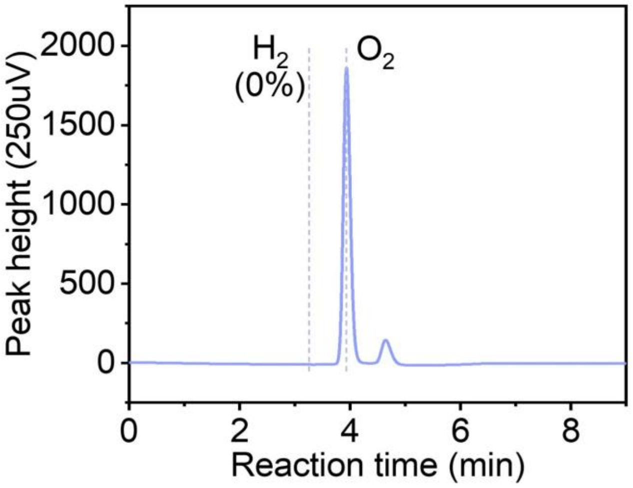

Supplementary Figure 19. The gas chromatography (GC) analyses of gaseous products collected in prototype device based on ZIF-350 electrode at 1 A cm^{-2}. Analysis of the gas products after the 2e-ORR test by gas chromatography (GC) revealed that undetectable H_2 was produced from side HER.

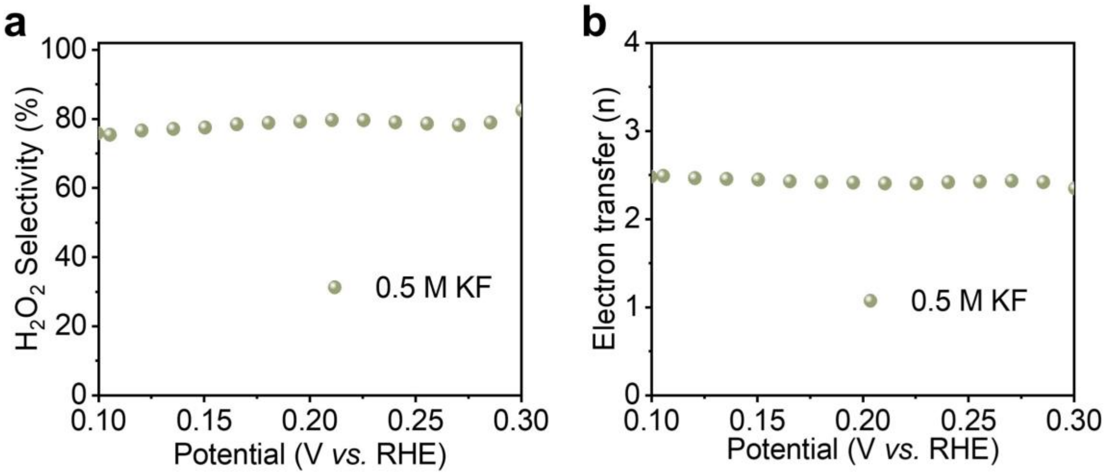

Supplementary Figure 20. Rotating ring-disk electrode (RRDE) test of ZIF-350 in air. a, H_2O_2 selectivity calculated on the basis of RRDE. **b.** Electron transfer number (n) calculated on the basis of RRDE.

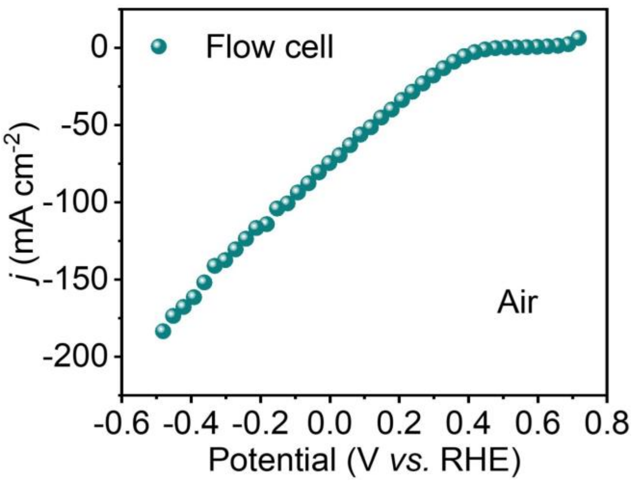

Supplementary Figure 21. LSV curves of ZIF-350 tested in a flow cell system in air. ZIF-350 electrode has exhibited a well-defined LSV profile in KF electrolyte, where the current density rapidly increases with negatively polarized potentials, suggesting the occurrence of oxygen reduction reaction (ORR) in air atmosphere.

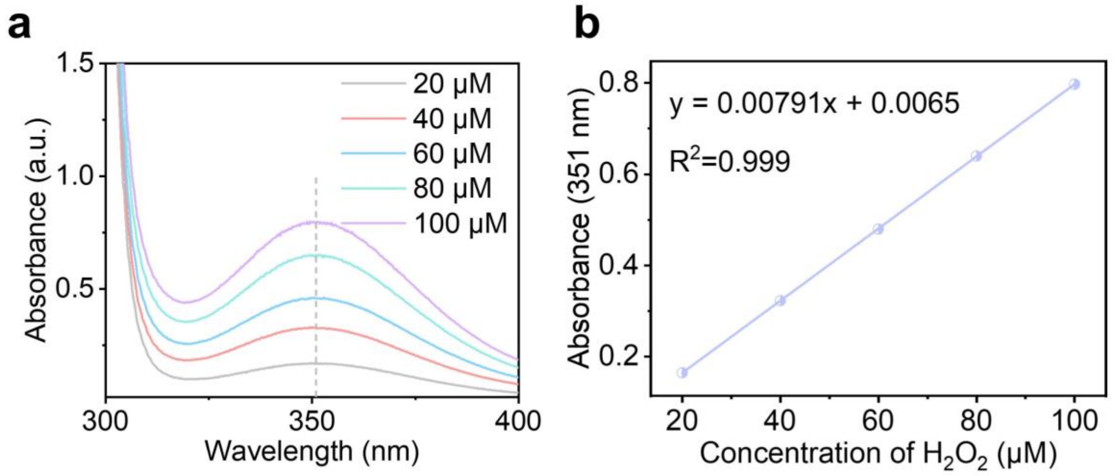

Supplementary Figure 22. Standard calibration curves for quantifying H_2O_2. a, UV-vis spectra of I_3^- solution with various concentrations. **b,** The corresponding standard curve. The H_2O_2 concentration was quantified by iodometric method. The UV-vis absorbance of the resulted mixture was measured at the wavelength of 351 nm.

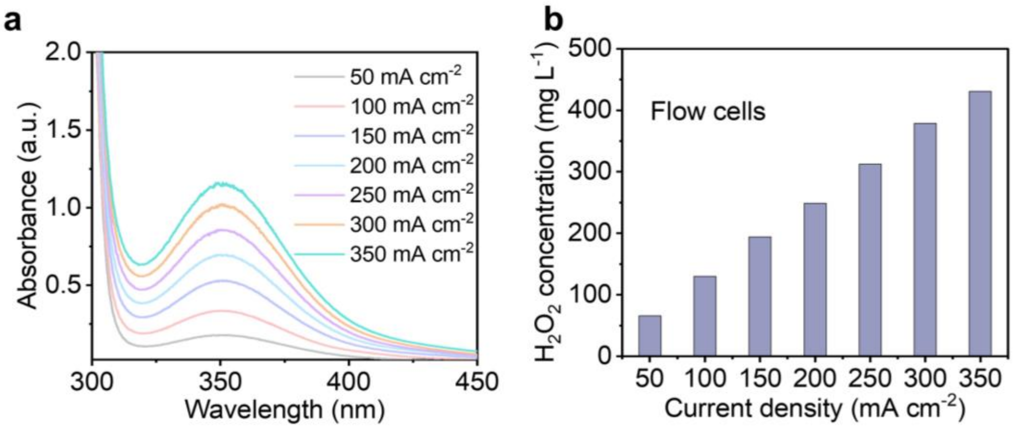

Supplementary Figure 23. UV-vis spectra curves of ZIF-350 tested in different current densities in flow cell system in air. The air-to-H_2O_2 performance of ZIF-350 in KF was examined by UV-vis spectra, showing the concentration of H_2O_2 increases almost linearly throughout whole current density window (65.7−431.1 mg L^{-1}).

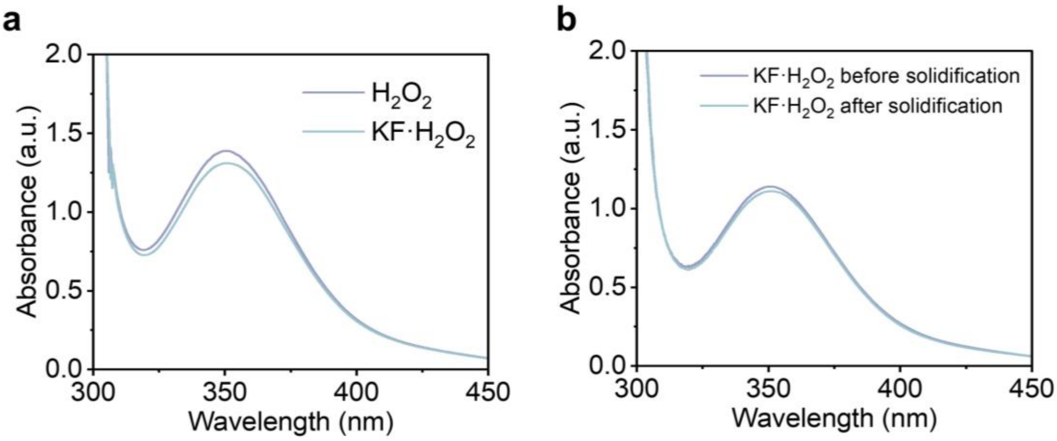

Supplementary Figure 24. Comparison experiments to determine the potential H_2O_2 loss during the solidification. a, UV-vis spectra of KF electrolyte with commercial H_2O_2 (500 mg L^{-1}) and KF electrolyte with an equivalent amount of solid KF·H_2O_2. **b**, UV-vis spectra of KF·H_2O_2 under electrolysis at 350 mA cm^{-2} before and after solidification.

Supplementary Note 5:

There might be potential H_2O_2 loss during the solidification, which causes data errors in determining the productivity of KF·H_2O_2. Consequently, we have added two comparison experiments to determine the potential H_2O_2 loss during the solidification as follows:

(1) We have prepared the following solutions: *i)* H_2O_2 in KF electrolyte (500 mg L^{-1}, the concentration in our electrosynthesis system); *ii*) solid KF·H_2O_2 in KF electrolyte (equivalent amount). As shown in Supplementary Fig. 24a, the UV-Vis spectra of both solutions were recorded using the KI-based colorimetric method (351 nm). The results showed identical absorption peaks with seldom peak intensity difference (3.05%).

This phenomenon indicates that UV-Vis quantification method can accurately reflects the KF·H_2O_2 concentration by using H_2O_2 in KF electrolyte.

(2) Further, we have electrochemically synthesized KF·H_2O_2 at the current density of 350 mA cm^{-2}. The concentration of as-produced KF·H_2O_2 in the electrolyte was determined by UV-Vis as 12.31 mmol L^{-1}. Next, the KF·H_2O_2-containing electrolyte was evaporated and dried under vacuum (40 °C) to obtain solid KF·H_2O_2. The mass of solid-state KF·H_2O_2 was determined by UV-Vis to be 12.06 mmol L^{-1}. The difference of KF·H_2O_2 before and after solidification was minor (2.03%, Supplementary Fig. 24b), indicating seldom loss of H_2O_2 during solidification and drying process.

Based on above discussions, we conclude that all of the electrochemical data is valid for use in our study.

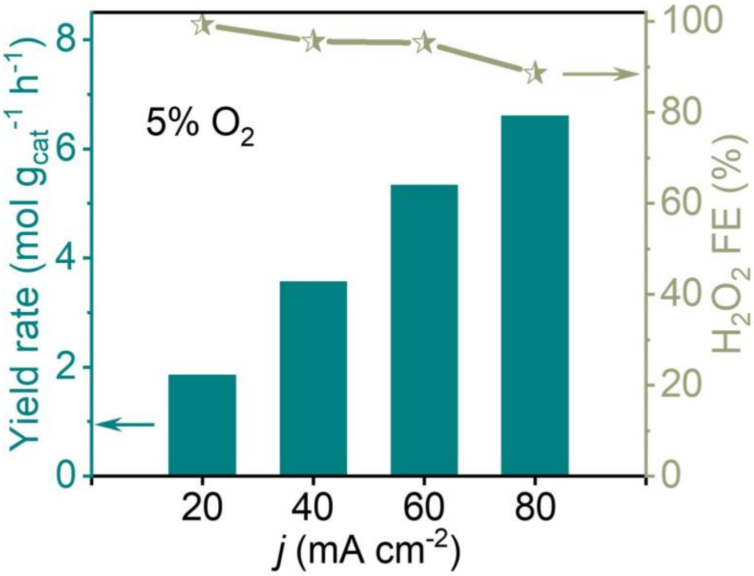

Supplementary Figure 25. The ORR activity for ZIF-350 catalyst in 5% O_2.

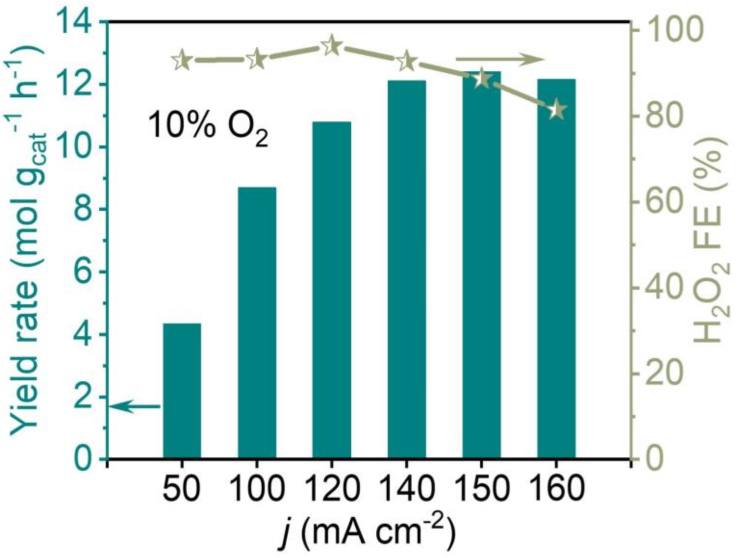

Supplementary Figure 26. The ORR activity for ZIF-350 catalyst in 10% O_2.

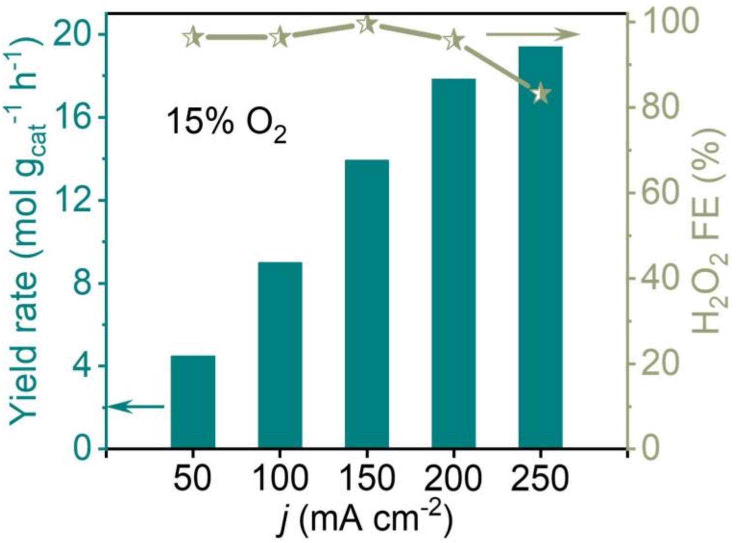

Supplementary Figure 27. The ORR activity for ZIF-350 catalyst in 15% O_2.

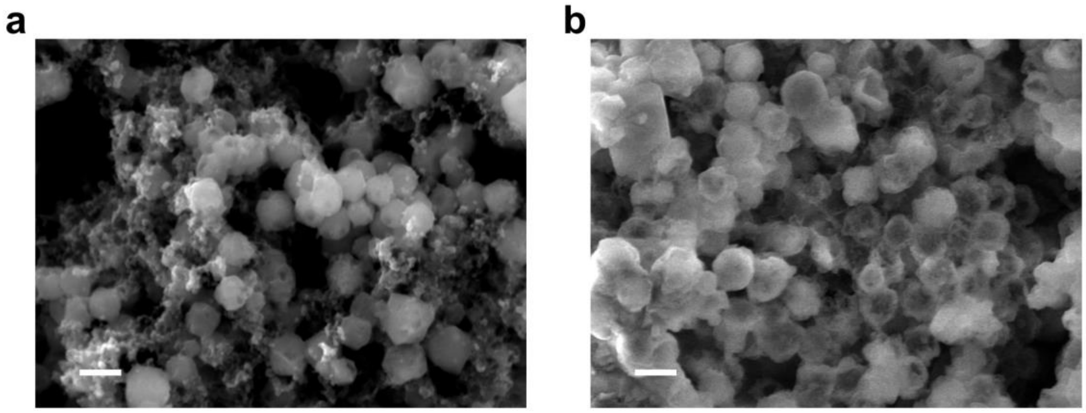

Supplementary Figure 28. The SEM images of ZIF-350 electrode before and after stability test. a, Before the test (scale bar: 1 μm). **b**, After the test (scale bar: 1 μm).

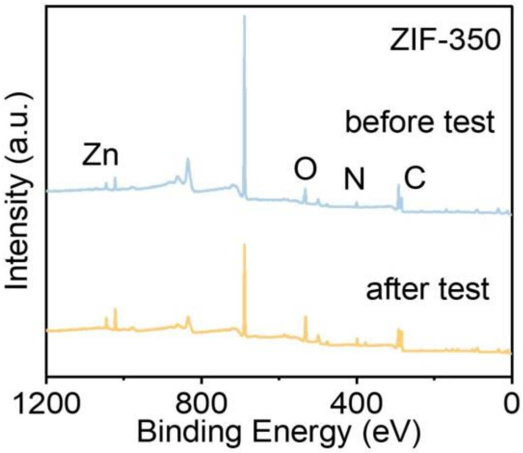

Supplementary Figure 29. The X-ray photoelectron spectra (XPS) of ZIF-350 electrode before and after stability test.

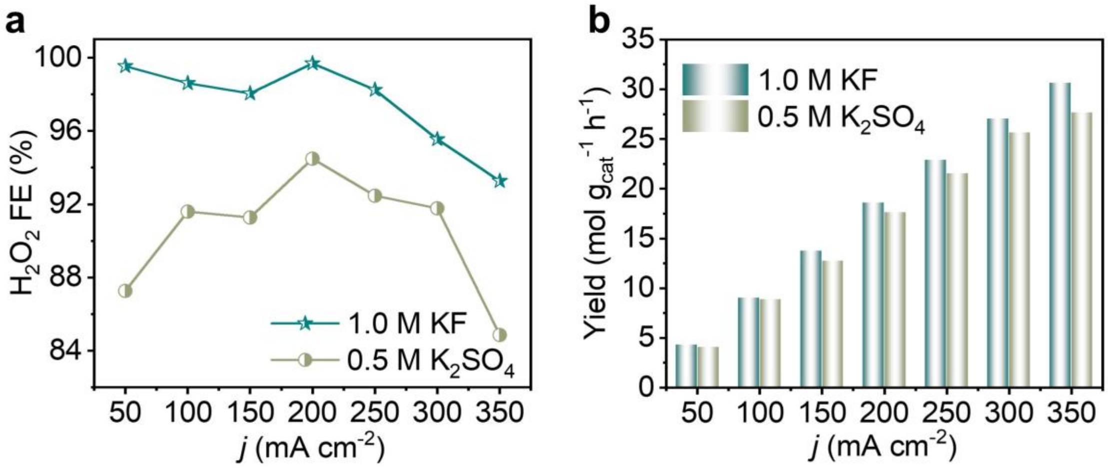

Supplementary Figure 30. The 2e-ORR performances of ZIF-350 in KF and K_2SO_4. a, H_2O_2 Faradauc efficiencies (FEs). **b.** H_2O_2 yield rates.

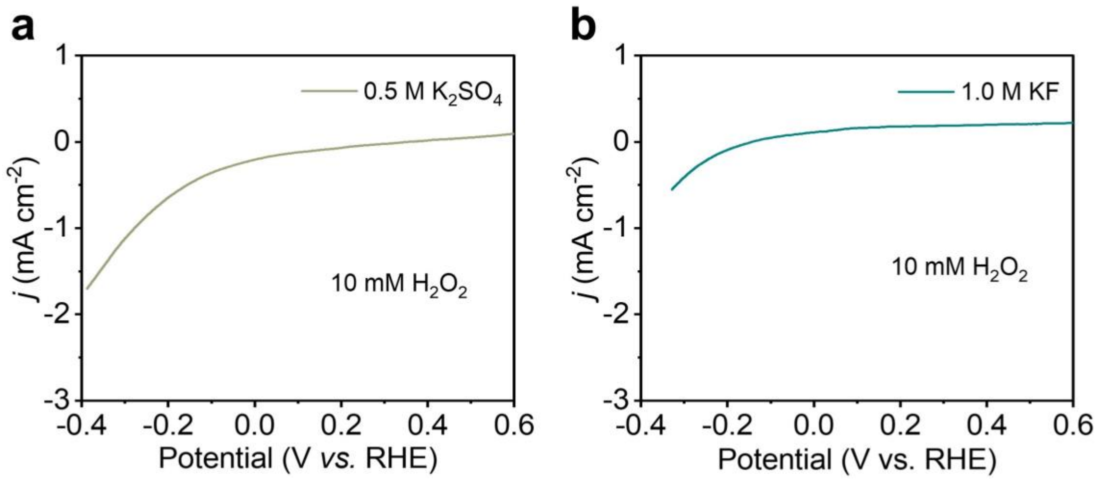

Supplementary Figure 31. H_2O_2 reduction polarization curves containing 10 mM H_2O_2 in different electrolytes. a, 0.5 M K_2SO_4. **b.** 1.0 M KF.

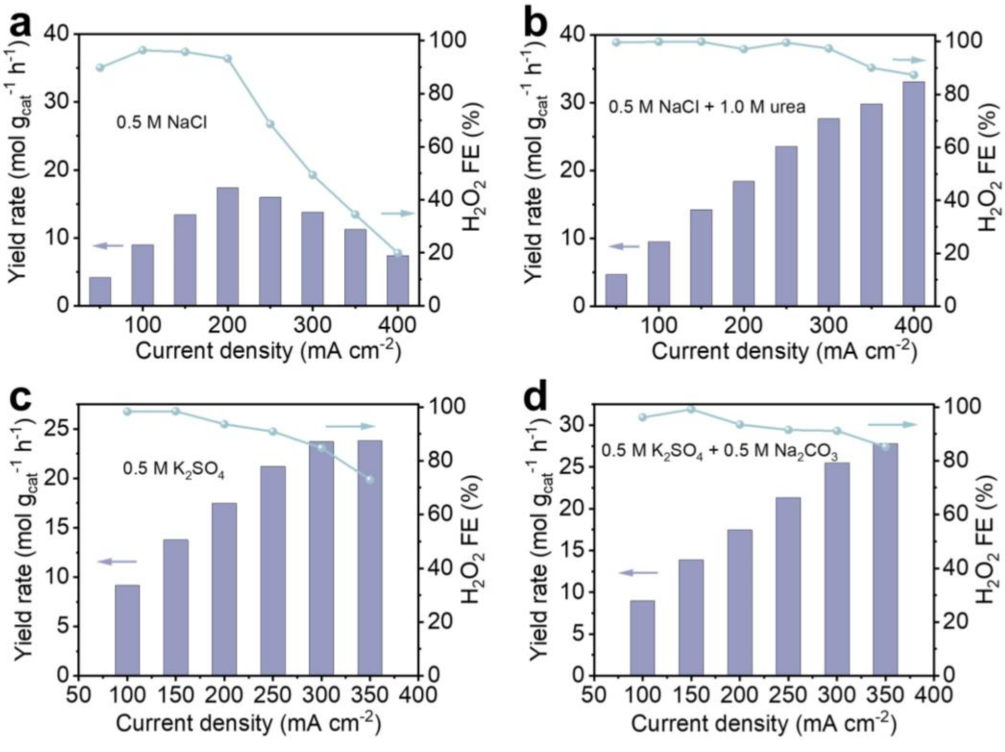

Supplementary Figure 32. The 2e-ORR performances in urea and Na_2CO_3. a, 0.5 M NaCl. **b**, 0.5 M NaCl with 1.0 M urea. **c**, 0.5 M K_2SO_4. **d**, 0.5 M K_2SO_4 with 0.5 M Na_2CO_3.

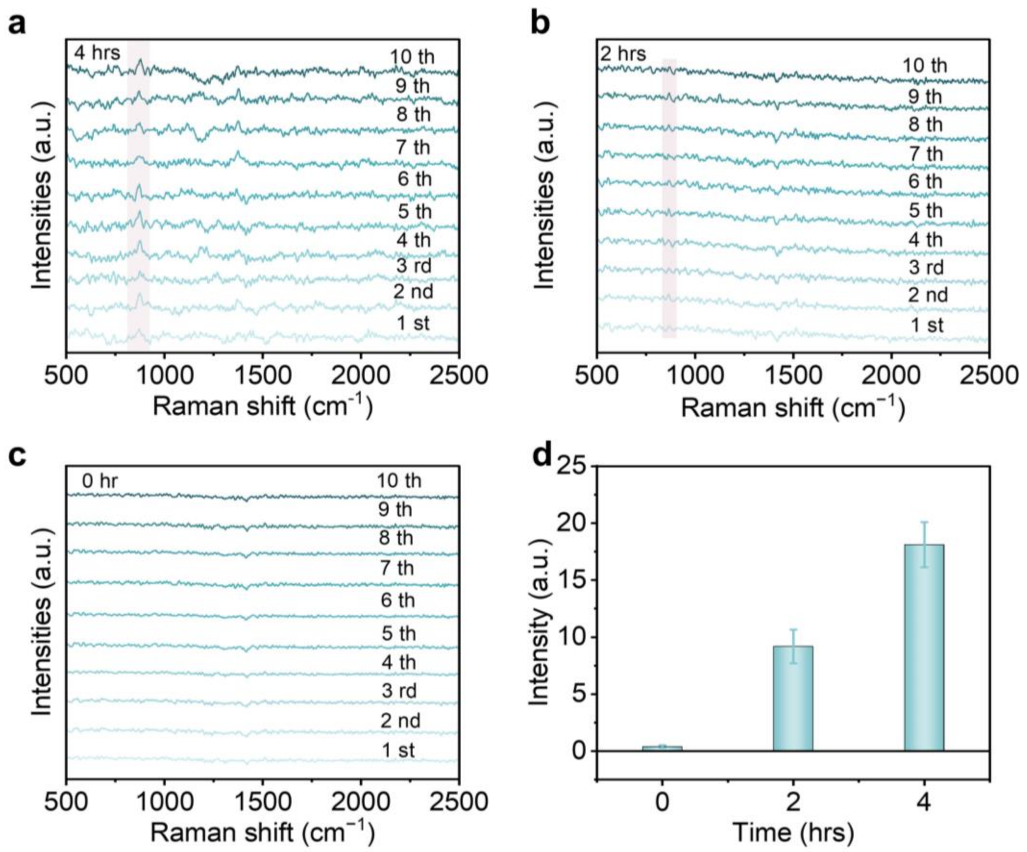

Supplementary Figure 33. Ten repetitive *operando* Raman tests for ZIF-350 in air under the same condition. **a**, 4 hrs. **b**, 2 hrs. **c**, 0 hr. **d**, The 858 cm^{-1} peak intensity with error bar in *operando* Raman test. Ten measurements were conducted for each data point with the error bars corresponding to the standard deviation.

Supplementary Note 6:

Additional ten repetitive tests were performed for *operando* Raman under the same condition of Supplementary Figure 32. The weak Raman signal is due to the harsh *operando* test condition[11]. In this work, the *operando* Raman only probes the signals of O-O stretching vibrations in H_2O_2 during catalytic processes. The scattered Raman signals are mainly focused on bond vibrations of adsorbed species (like $*O_2$ and *OOH in ORR) in electrolyte, which are known to be very weak as comparison to

bulk materials. To confirm the accuracy of the experimental results, Raman spectrometer software is used to directly determine the peak positions and intensities. On this basis, the Raman information of H_2O_2 in KF are compared at different durations. By comparing the Raman vibration of ZIF-350 at different electrolysis duraitons[12, 13], we conclude the intensity of *OOH peak increased consistently with elongated reaction duration, proving the validity of our operando synthesis condition.

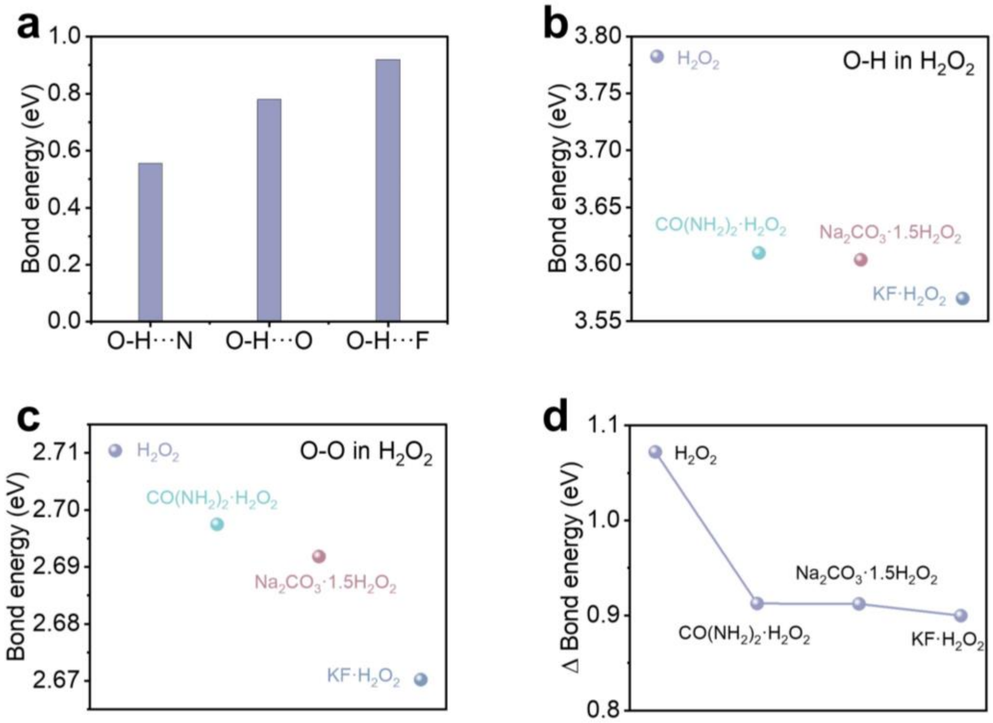

Supplementary Figure 34. Bond energies of isolated H_2O_2 and H_2O_2 in peroxosolvates. **a**, Bond energies of O-H···N/O/F. **b**, Bond energies of O-H. **c**, Bond energies of O-O. **d**, Bond energy difference between O-H and O-O bonds.

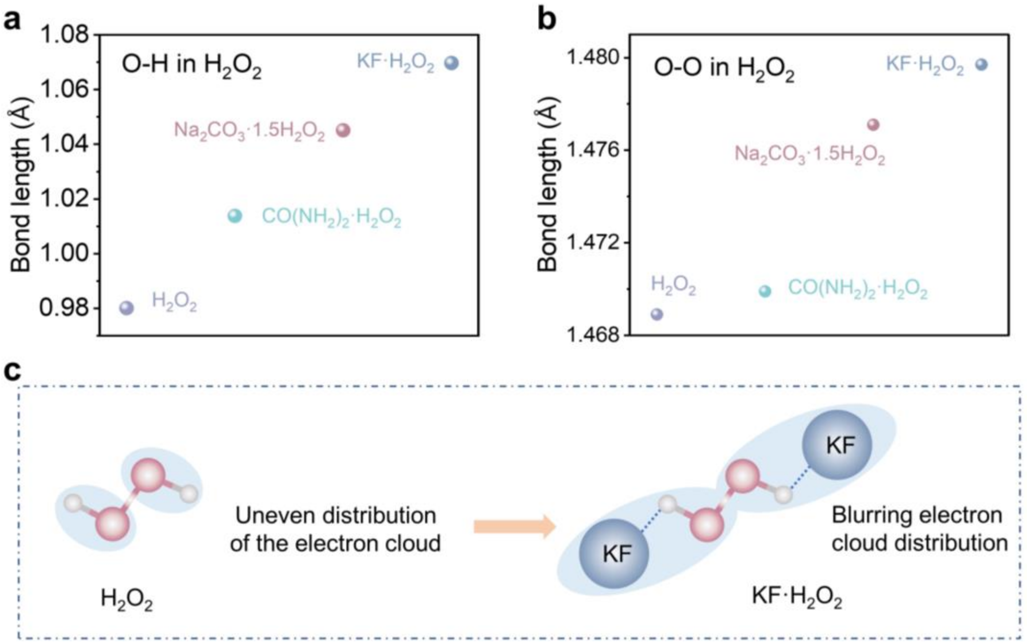

Supplementary Figure 35. Bond length of isolated H_2O_2 and H_2O_2 in peroxosolvates. **a**, Bond length of O-H in H_2O_2. **b**, Bond length of O-O in H_2O_2. **c**, Schematic of the stabilization of H_2O_2 by blurring effect.

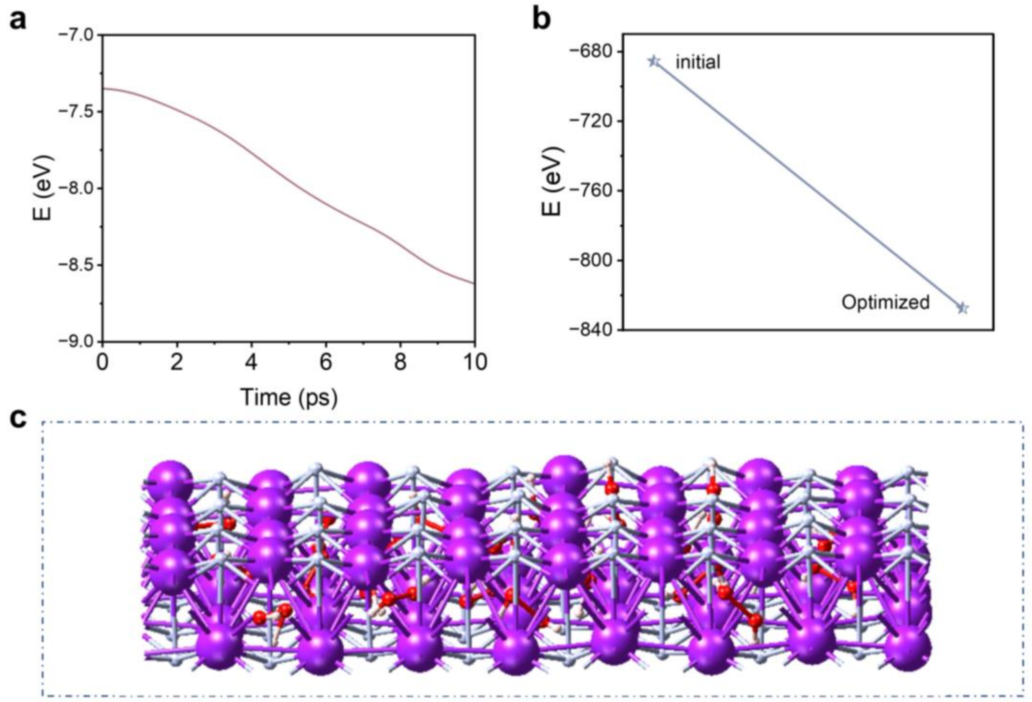

Supplementary Figure 36. Molecular dynamics (MD) simulations of the interaction between KF and H_2O_2 (The red, white, purple, and blue atoms denote oxygen, hydrogen, potassium, and fluorine, respectively). a, Kinetic energies. b, Thermodynamic energies. c, The optimized model of KF and H_2O_2.

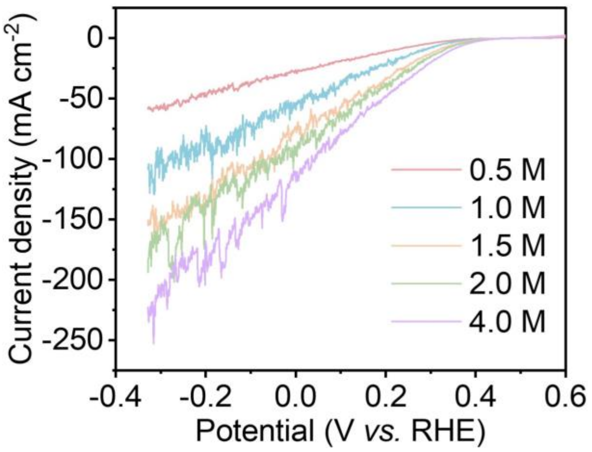

Supplementary Figure 37. LSV curves tested in different concentrations of KF electrolytes.

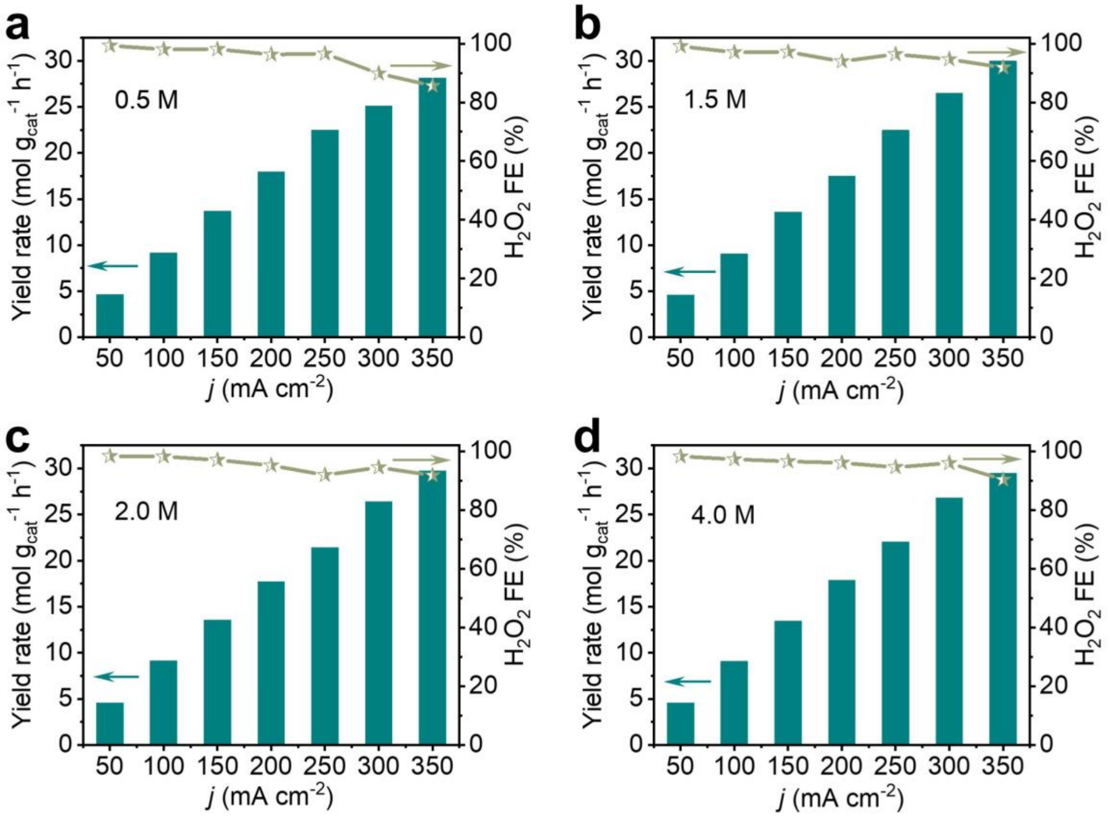

Supplementary Figure 38. 2e-ORR performances in different KF concentrations.

a, 0.5 M. **b**, 1.5 M. **c**, 2.0 M. **d**, 4.0 M.

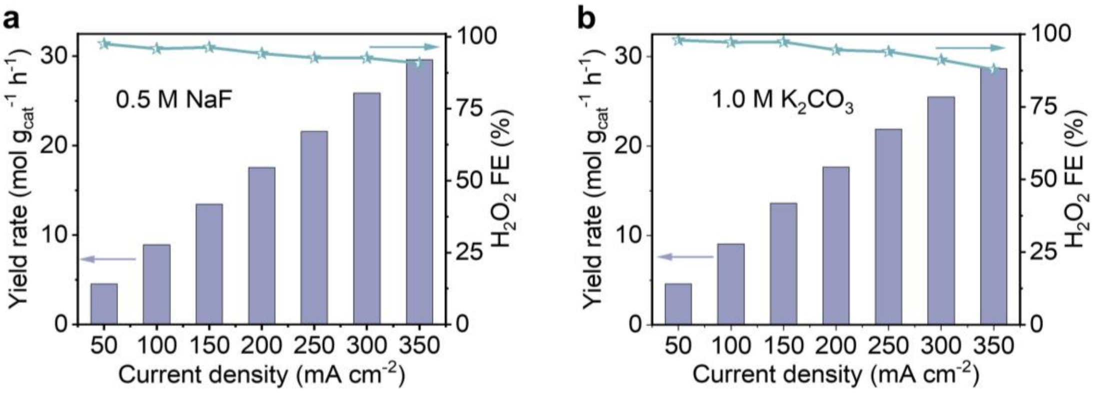

Supplementary Figure 39. The 2e-ORR performances of ZIF-350 in NaF and K_2CO_3 electrolytes. a, 0.5 M NaF. **b**, 1.0 M K_2CO_3.

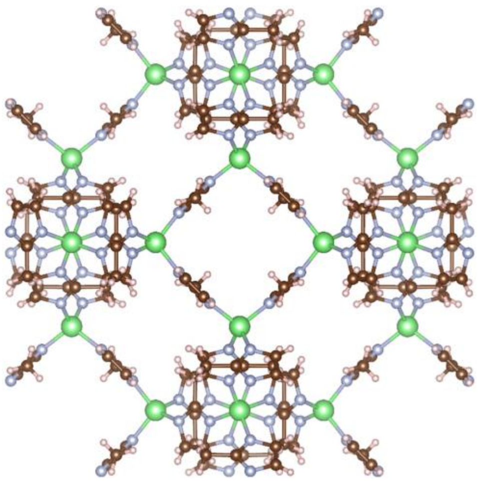

Supplementary Figure 40. The unit structure of ZIF in this work. The red, white, purple, brown and blue atoms denote O, H, C, and N, respectively.

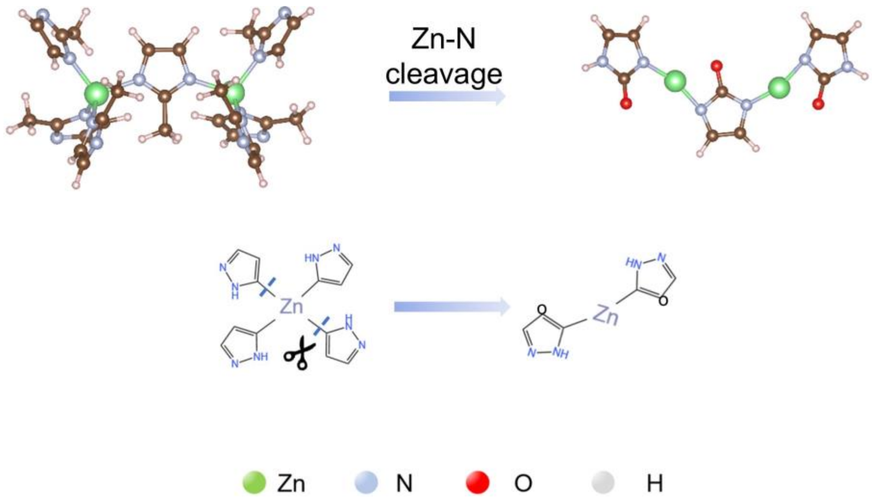

Supplementary Figure 41. The optimized structures for ZIF and ZIF-350. The red, white, purple, brown and blue atoms denote O, H, C, and N, respectively.

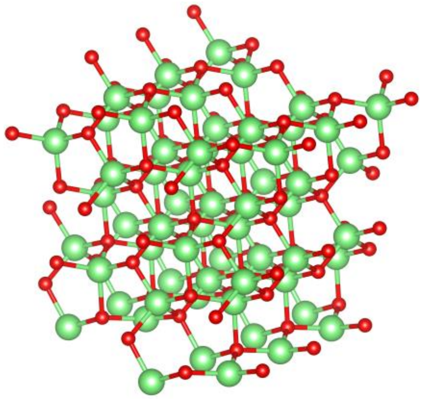

Supplementary Figure 42. The optimized structure of ZIF-600.

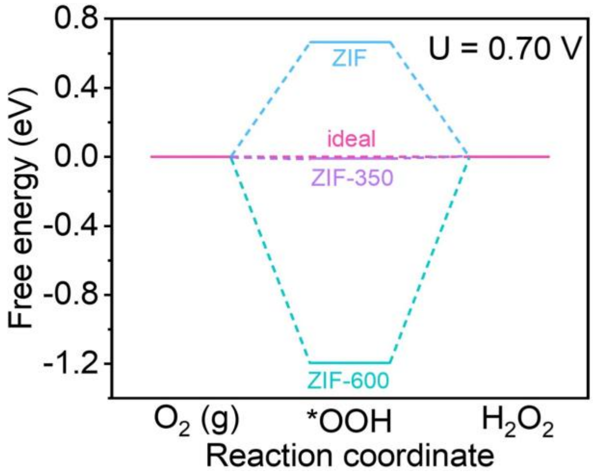

Supplementary Figure 43. Free-energy diagrams for 2e-ORR pathway at 0.7 V *vs.* RHE.

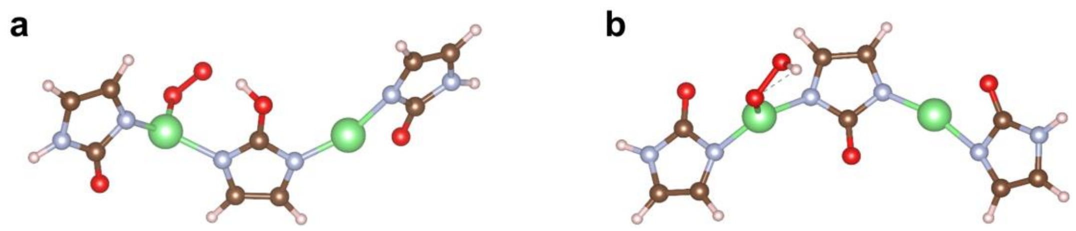

Supplementary Figure 44. The structure of intermediate for ZIF-350 following E-R and L-H pathway. a, the structure of *O_2-*H intermediate for the reference ZIF-350 following L-H pathway. **b**, the structure of *OOH intermediate for the reference ZIF-350 following E-R pathway.

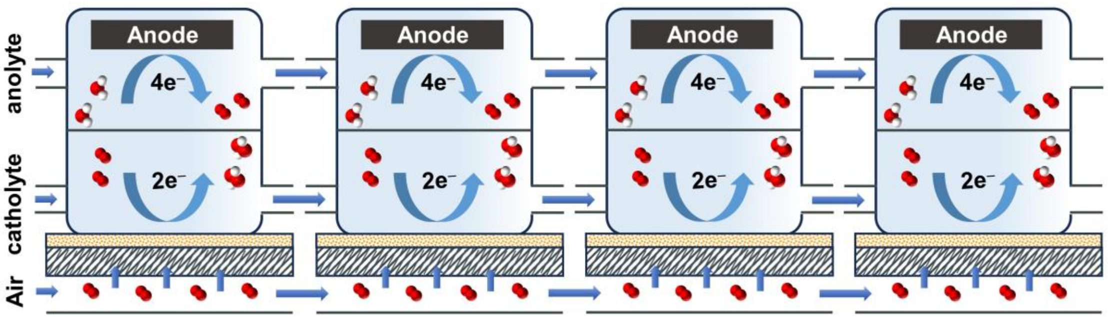

Supplementary Figure 45. Schemetic conventional system: four flow-type cells connecting electrolyte in tandem and an electrical circuit in parallel.

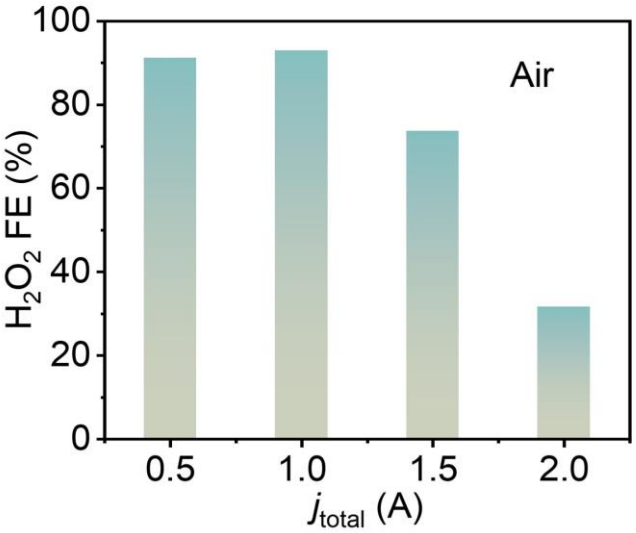

Supplementary Figure 46. The 2e-ORR Performances in conventional stacks.

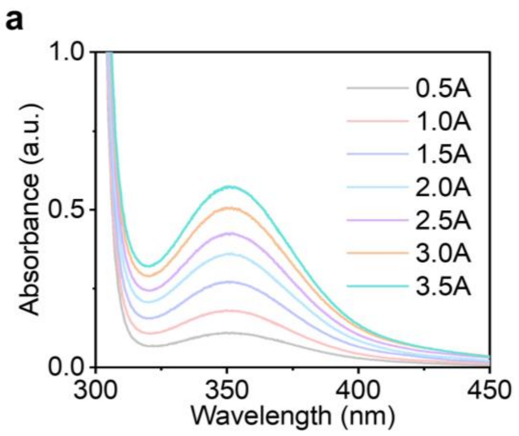

Supplementary Figure 47. UV-vis spectra curves of ZIF-350 tested in the present system under air. ZIF-350 maintains exceptional 2e-ORR performance even at ampere-level currents. UV-vis spectra confirm the H_2O_2 concentration increasing linearly with current density (173.6−1104.4 mg L^{-1}).

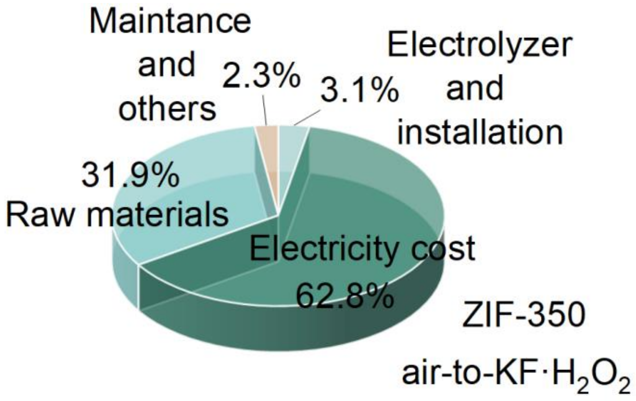

Supplementary Figure 48. The proportions of $KF\cdot H_2O_2$ production cost for ZIF-350 in atmospheric air.

3. Supplementary Tables

Supplementary Table 1. Number of cycles for KF to store and release H_2O_2. In a typical cycle for KF, the low-temperature heating of $KF{\cdot}H_2O_2$ releases H_2O_2, where KF is left for further storage.

**Cycle number**	**Mass of $KF{\cdot}H_2O_2$ (g)**
1	1.59
10	1.57
20	1.58
30	1.58
40	1.57
50	1.57
60	1.58
70	1.58
80	1.59
90	1.59
100	1.58

Supplementary Table 2. Stability of $KF \cdot H_2O_2$.

**Days of H_2O_2 storage**	**Mass of $KF \cdot H_2O_2$ (g)**
0	1.69
14	1.68
28	1.67
42	1.67
56	1.66
70	1.65
84	1.65
98	1.63
112	1.62
126	1.61
140	1.60
160	1.60

Supplementary Table 3. Extended X-ray absorption fine structure (EXAFS) fitting parameters for Zn K-edge.

**Sample**	**Path**	**CN**	**R (Å)**	**σ^2 (Å^2)**	**ΔE_0 (eV)**	**R-factor**
Zn foil	Zn-Zn	6.6	2.65	12.45	0.072	0.007
ZnO	Zn-O	4.0	1.95	3.45	4.471	0.008
ZIF-350	Zn-N	2.4	1.97	6.35	4.76	0.010

NOTE: CN: coordination number; R: bond lengths between central atoms and surrounding coordination atoms; σ^2: Debye-Waller factor to account for both thermal and structural disorders; ΔE_0: the difference of the zero kinetic energy value between the sample and theoretical model; R-factor is used to measure the goodness of the fitting.

Supplementary Table 4. Zn percentages in ZIF-350 electrode after 80 hrs-stability test.

Material	Zn Atomic %
ZIF-350	4.09

Supplementary Table 5. The comparison of Faradaic efficiencies (FE) of different electrolytes with ZIF-350 electrode for air-to-H_2O_2 conversion.

Current densities (mA cm^{-2})	1.0 M KF (%)	0.5 M K_2SO_4 (%)
50	99.54	87.27
100	98.61	91.60
150	98.05	91.28
200	99.69	94.49
250	98.25	92.47
300	95.55	91.78
350	93.27	84.86

Supplementary Table 6. The comparison of H_2O_2 yield rates in different electrolytes with ZIF-350 electrode for air-to-H_2O_2 conversion.

Current densities ($mA\ cm^{-2}$)	1.0 M KF ($mol\ g_{cat}^{-1}\ h^{-1}$)	0.5 M K_2SO_4 ($mol\ g_{cat}^{-1}\ h^{-1}$)
50	4.64	4.07
100	9.19	8.89
150	13.71	12.76
200	17.56	17.11
250	22.05	21.55
300	26.72	25.66
350	30.64	27.68

Supplementary Table 7. Free energies G (eV) for the adsorbates on the Zn site of ZIF-350 at T = 298 K.

**Adsorbate**	**ZIF-350**
*OOH	4.21
*O	3.94
*OH	0.99
*O_2-*H	5.51

Supplementary Table 8. Adsorption energy ΔE_{ads} (eV) of KF on adsorbates.

**Adsorbate**	**KF**
H_2O	−0.50
H_2O_2	−0.90

Supplementary Table 9. Comparison of the electrochemical air-to-H_2O_2 conversion activities of ZIF-350 with state of-the-art catalysts. LM: loading mass (mg cm^{-2}); E: electrolyte; j: current (A); Yield rate: mol g_{cat}^{-1} h^{-1}; FE: Faraday efficiency (%).

Catalyst	E	A	LM	j	FE	Yield rate	Stability	Reference[8, 14-21]
ZIF-350	1.0 M KF	16	0.2	3.5	89.62	58.47	50 h@1 A	This work
ER-ZnO	0.6 M K_2SO_4	1	0.2	0.3	89.3	4.88	100 h@0.2 A	Nat Commun. 15, 4157 (2024)
N, S-TCNTs	1.0 M KOH	1	0.2	0.35	93.0	15.19	200 h@0.1 A	Adv. Mater. 2023, 35, 2303905
Co HSACs	0.5 M KOH	1	0.5	0.3	90	5	25 h@0.3 A	Nat Commun. 14, 1426 (2023)
In SAs/NSBC	0.1 M KOH	7	0.2	0.63	77.34	9.09	12 h@0.6 V_{RHE}	Angew. Chem. Int. Ed. 2022, 61, e202117347
N, O-CNTs	1.0 M KOH	1	2	0.048	95	2.65	24 h@~48 mA	Adv. Sci. 2022, 9, 2201421
PD/N-C	0.1 M $HClO_4$	4	/	0.1	89	10.74	240 h@−0.6 V_{RHE}	J. Am. Chem. Soc. 2023, 145, 11589−11598
CoSP/MWCNTs	0.05 M Na_2SO_4	30	2	0.8	60	6.36	/	Chem. Eng. J. 2020, 379, 122417
Co-NC/Mxenes	0.5 M H_2SO_4	4	0.2	0.5	90	3.02	40 h@0.1 V_{RHE}	Appl. Catal. B Environ. 2022, 317, 121737
CoPc/CNT	0.1 M H_2SO_4	12	0.4	0.48	/	17.81	8 h@40 mA	Chin. J. Catal. 2022, 43 (5),

								1238−1246
FCB	SEC	4	0.46	0.8	61.5	42.32	100 h@120 mA	Science 366, 226-231 (2019)

Supplementary Table 10. Average mass loadings of ZIF-350 catalysts. Actual test area is 1 cm^2.

m (carbon fiber paper)/ mg	m (ZIF-350 on carbon fiber paper)/ mg	Area/cm^{-2}	Mass loading/mg cm^{-2}
33.47	34.04	3.0	0.190
35.12	35.74	3.0	0.207
36.89	37.43	3.0	0.180
34.25	34.94	3.0	0.230
37.56	38.14	3.0	0.193
32.15	32.80	3.0	0.217
35.78	36.38	3.0	0.200
33.91	34.46	3.0	0.183
36.23	36.89	3.0	0.220
34.67	35.26	3.0	0.197
average			0.202

Supplementary Note 7:

The catalyst loading was determined by the following method:

$$\text{Mass loading} = (m_2 - m_1)/A$$

where m_1 is the mass of carbon fiber paper, m_2 is the mass of ZIF-350 on carbon fiber paper, A is the geometric of carbon fiber paper.

To obtain a more data, we repeated the material synthesis for ten times, and then calculate the average mass loading for ZIF-350.

4. Supplementary References

1. Perdew J. P., Burke K., Ernzerhof M. Generalized gradient approximation made simple. *Phys. Rev. Lett.* **77**, 3865−3868 (1996).
2. Perdew J. P., Burke K., Wang Y. Generalized gradient approximation for the exchange-correlation hole of a many-electron system. *Phys. Rev. B* **54**, 16533−16539 (1996).
3. Blöchl P. E. Projector augmented-wave method. *Phys. Rev. B* **50**, 17953−17979 (1994).
4. Kresse G., Joubert D. From ultrasoft pseudopotentials to the projector augmented-wave method. *Phys. Rev. B* **59**, 1758−1775 (1999).
5. Nami H., Rizvandi O. B., Chatzichristodoulou C., Hendriksen P. V., Frandsen H. L. Techno-economic analysis of current and emerging electrolysis technologies for green hydrogen production. *Energ. Convers. Manage.* **269**, 116162 (2022).
6. Zhang X., Fang Z. W., Zhu P., Xia Y., Wang H. T. Electrochemical regeneration of high-purity CO_2 from (bi) carbonates in a porous solid electrolyte reactor for efficient carbon capture. *Nat. Energy*, **10**, 55–65 (2025).
7. Wang X., *et al.* Efficient electrosynthesis of n-propanol from carbon monoxide using a Ag-Ru-Cu catalyst. *Nat. Energy* **7**, 170–176 (2022).
8. Huang Q., *et al.* Single-zinc vacancy unlocks high-rate H_2O_2 electrosynthesis from mixed dioxygen beyond Le Chatelier principle. *Nat. Commun.* **15**, 4157 (2024).
9. Jung E., *et al.* Atomic-level tuning of Co-N-C catalyst for high-performance electrochemical H_2O_2 production. *Nat. Mater.* **19**, 436–442 (2020).
10. Lee B.-H., *et al.* Supramolecular tuning of supported metal phthalocyanine catalysts for hydrogen peroxide electrosynthesis. *Nat. Catal.* **6**, 234–243 (2023).

11. Yu Z., *et al.* Interfacial engineering of heterogeneous molecular electrocatalysts using ionic liquids towards efficient hydrogen peroxide production. *Chinese J. Catal.* **43**, 1238–1246 (2022).
12. Dong J.-C., *et al.* Direct in situ Raman spectroscopic evidence of oxygen reduction reaction intermediates at high-index Pt (hkl) surfaces. *J. Am. Chem. Soc.* **142**, 715–719 (2020).
13. Zhao Y. L., *et al.* Identification of M-NH_2-NH_2 intermediate and rate determining step for nitrogen reduction with bioinspired sulfur-bonded FeW catalyst. *Angew. Chem. Int. Ed.* **60**, 20331–20341 (2021).
14. Alcaide F., Álvarez G., Guelfi D. R. V., Brillas E., Sirés I. A stable CoSP/MWCNTs air-diffusion cathode for the photoelectro-Fenton degradation of organic pollutants at pre-pilot scale. *Chem. Eng. J.* **379**, 122417 (2020).
15. Fan W. J., *et al.* Rational design of heterogenized molecular phthalocyanine hybrid single-atom electrocatalyst towards two-electron oxygen reduction. *Nat. Commun.* **14**, 1426 (2023).
16. Huang X., *et al.* Nb_2CT_x MXenes functionalized Co-NC enhancing electrochemical H_2O_2 production for organics degradation. *Appl. Catal. B: Environ.* **317**, 121737 (2022).
17. Long Y. D., *et al.* Tailoring the atomic-local environment of carbon nanotube tips for selective H_2O_2 electrosynthesis at high current densities. *Adv. Mater.* **35**, 2303905 (2023).
18. Xia C., Xia Y., Zhu P., Fan L., Wang H. T. Direct electrosynthesis of pure aqueous H_2O_2 solutions up to 20% by weight using a solid electrolyte. *Science* **366**, 226–231 (2019).
19. Xu S. H., *et al.* Synergistic effects in N, O-comodified carbon nanotubes boost highly selective electrochemical oxygen reduction to H_2O_2. *Adv. Sci.* **9**, 2201421 (2022).
20. Zhang C., *et al.* A pentagonal defect-rich metal-free carbon electrocatalyst for boosting acidic O_2 reduction to H_2O_2 production. *J. Am. Chem. Soc.* **145**, 11589–11598 (2023).

21. Zhang E. H., *et al.* Engineering the local atomic environments of indium single-atom catalysts for efficient electrochemical production of hydrogen peroxide. *Angew. Chem. Int. Ed.* **61**, e202117347 (2022).
