# Supplementary material for: Electronic structure blurring-mediated solid-state H2O2 electrosynthesis with high productivity
Source: Nat Commun. 2025 Nov 24;16:10360. doi: 10.1038/s41467-025-65335-5 (PMC12645009; doi:10.1038/s41467-025-65335-5)
Supplement: Supplementary file 2 — Description of Additional Supplementary Files [file 41467_2025_65335_MOESM2_ESM.pdf]

Description of Additional Supplementary Files:

Supplementary Data 1: This txt file provide the DFT calculation model for the optimized structures of ZIF-350

Supplementary Data 2: This txt file provide the DFT calculation model for the optimized structures of ZIF - 350-*OOH

Supplementary Data 3: This txt file provide the DFT calculation model for the optimized structures of ZIF-350 LH (the structure of *O2-*H intermediate for the reference ZIF-350 following L-H pathway)
